# Repetitive Religious Chanting Modulates the Late-Stage Brain Response to Fear- and Stress-Provoking Pictures

**DOI:** 10.3389/fpsyg.2016.02055

**Published:** 2017-01-10

**Authors:** Junling Gao, Jicong Fan, Bonnie W. Wu, Georgios T. Halkias, Maggie Chau, Peter C. Fung, Chunqi Chang, Zhiguo Zhang, Yeung-Sam Hung, Hinhung Sik

**Affiliations:** ^1^Centre of Buddhist Studies, The University of Hong KongHong Kong, Hong Kong; ^2^Department of Electrical and Electronic Engineering, The University of Hong KongHong Kong, Hong Kong; ^3^Department of Electronic Engineering, City University of Hong KongHong Kong, Hong Kong; ^4^School of Biomedical Engineering, Shenzhen UniversityShenzhen, China

**Keywords:** event-related potential, negative bias, late positive potential, source analysis, stress, chanting Amitābha Buddha, religious schema, emotion regulation

## Abstract

Chanting and praying are among the most popular religious activities, which are said to be able to alleviate people’s negative emotions. However, the neural mechanisms underlying this mental exercise and its temporal course have hardly been investigated. Here, we used event-related potentials (ERPs) to explore the effects of chanting the name of a Buddha (Amitābha) on the brain’s response to viewing negative pictures that were fear- and stress-provoking. We recorded and analyzed electroencephalography (EEG) data from 21 Buddhists with chanting experience as they viewed negative and neutral pictures. Participants were instructed to chant the names of Amitābha or Santa Claus silently to themselves or simply remain silent (no-chanting condition) during picture viewing. To measure the physiological changes corresponding to negative emotions, electrocardiogram and galvanic skin response data were also collected. Results showed that viewing negative pictures (vs. neutral pictures) increased the amplitude of the N1 component in all the chanting conditions. The amplitude of late positive potential (LPP) also increased when the negative pictures were viewed under the no-chanting and the Santa Claus condition. However, increased LPP was not observed when chanting Amitābha. The ERP source analysis confirmed this finding and showed that increased LPP mainly originated from the central-parietal regions of the brain. In addition, the participants’ heart rates decreased significantly when viewing negative pictures in the Santa Claus condition. The no-chanting condition had a similar decreasing trend although not significant. However, while chanting Amitābha and viewing negative pictures participants’ heart rate did not differ significantly from that observed during neutral picture viewing. It is possible that the chanting of Amitābha might have helped the participants to develop a religious schema and neutralized the effect of the negative stimuli. These findings echo similar research findings on Christian religious practices and brain responses to negative stimuli. Hence, prayer/religious practices may have cross-cultural universality in emotion regulation. This study shows for the first time that Buddhist chanting, or in a broader sense, repetition of religious prayers will not modulate brain responses to negative stimuli during the early perceptual stage, but only during the late-stage emotional/cognitive processing.

## Introduction

Religion and spirituality have long co-existed in Western and Eastern civilizations. One essential function of religion is that it provides a way for individuals to cope during hardship, such as after the death of a loved one or when faced with a debilitating, chronic illness (McIntosh, 1995; Taylor, 2001). Among a variety of religious activities, praying and chanting are quite common forms of practice in major religions including Christianity, Judaism, Islam, Hinduism, and Buddhism, etc. (McKim, 2016). In Southeast Asia, chanting Amitābha Buddha is among the most common religious practices, and it dates back to Indian Buddhism^1^. Buddhist practitioners of the Pure Land School have integrated the chanting of the name of Amitābha throughout their daily activities (Halkias, 2013a). According to the religious beliefs of Pure Land practitioners, the consistent chanting of Amitābha is a mind-training technique that can “hamper conceptual proliferation, quiet the discursive mind and the elimination of one’s wanton grasping after the fleeting impressions of the senses” (Halkias, 2013b).

Despite the major dominance of religious praying/chanting in daily life, relatively few studies have probed into this research area with objective measures. Some behavioral studies have found that religious prayer or chanting helps followers to cope with bereavement or other negative events in life (McIntosh, 1995; Wortmann and Park, 2008). One recent EEG study among Christians and Muslims shows increased alpha waves during prayers, indicating a state of relaxation and mental concentration (Doufesh et al., 2012). Other studies using conventional subjective measures have found that mantra recitation (repetition of a specific sound) may have a healing effect on late-stage cancer patients, as measured by the psychological questionnaires and physical examinations (Khalsa et al., 2009; Jain, 2014). However, these studies lack the precision needed to delineate the neural correlates underlying the effect of religious chanting/praying. Technologically, current advances in neuroscience have enabled us to examine brain responses to negative contexts with high temporal resolution. Previous event-related potential (ERP) study has found that negative emotion processing occurs at different locations along several time courses. The time courses can largely be distinguished by attention allocation in the early stage and cognitive evaluation at the later stages (Huang and Luo, 2006).

In this study, negative visual stimuli were used to test the influence of religious chanting on early- and late-stage neural information processing. It has been argued that evolution has made us more susceptible to negative events and, hence, increased our chance of survival. This phenomenon is referred to as negativity bias, which can widely influence cognitive and psychological characteristics including attention, learning, memory, moral judgment, and contagion (Rozin and Royzman, 2001). On the other hand, strong negative bias and negative proliferation could make the individuals more vulnerable to depression (Rude et al., 2002), as these individuals tend to overreact and inflict greater psychological anguish on themselves (Hanson, 2013).

Negativity bias occurs in different brain processing stages, including attention at the earlier stage and evaluation and reaction readiness at the later stages (Huang and Luo, 2006). For example, when compared to pleasantly valenced pictures, viewing of unpleasantly valenced pictures engages more focal attentional processes (Smith et al., 2003). While discerning the neural processing of negativity bias, P1 and N1 of ERP components are found to relate to early sensory and perceptual processing in the extrastriate visual cortex, and these components are sensitive to the graphic physical features in negative stimuli. Furthermore, the N1 component elicited by highly arousing negative pictures is robust and resistant to habituation as compared to other categories of pictures (Carretie et al., 2003). Negative pictures also elicit greater LPP than neutral pictures and positive pictures with equal arousal levels (Ito et al., 1998).

In addition, negative events also affect the autonomic nervous system (ANS) which controls the fight-or-flight response (Schmidt and Thews, 1989). The ANS can influence cognition and emotion, and vice versa. Physiological changes such as heart rate and galvanic skin response (GSR), among others, correlate with internal emotional responses (Libby et al., 2012). For example, the response of disgust becomes more intense at the systolic stage of heart beat, which highlights a close coupling of emotional processes and visceral activities (Gray et al., 2012). Moreover, emotions and religious beliefs are closely related, with emotion playing a substantial role in cultural settings and in a variety of religious contexts (Corrigan, 2008).

Previous studies have demonstrated that the effect of emotion and negative events may be modulated by participating in behavior therapy and practice of mindfulness (Fassbinder et al., 2016). Evidence shows that background music can also modulate the brain response to negative events to some degree (Waldie et al., 2013). Consistent with these works, Buddhist doctrine also suggests that a well-trained practitioner may modulate the mind’s response to negative events. According to the *Sallatha Sutta (The Arrow Sutra)*, a well-trained practitioner and an untrained layperson would both experience the initial feeling of pain in their primary states when they come across a harmful event, as if hit by an “arrow” (the “first arrow”), and this painful experience is unavoidable (Bhikkhu, 2013). While an untrained layperson experiences these unavoidable feelings, he or she may further develop negative emotions such as distress, despair, worry, etc. The proliferation of these secondary, often avoidable negative emotions, may subject the layperson to experience unnecessary emotional pain, analogous to being pierced by a “second arrow” (Sik, 2011). However, a well-trained practitioner would not react to these negative events with additional suffering; it is as if they would only be hit by a first arrow, but avoided the suffering of the second one (Sik, 2011). Base on this Buddhist understanding of two arrows of suffering, we theorize that during the processing of negative information, religious practice such as chanting would not affect the early neural processing of N1 (early stage attentional processing) but only affect the late-stage processing of LPP (later stage attentional processing) which appears to be elicited specifically by the presence of emotional information (Anderson and Stanford, 2012).

In this study, ERP was used to investigate whether religious chanting could affect negative information processing. We hypothesized that chanting Amitābha Buddha might alter the brain’s response to negative pictures during late-stage processes involved in cognition when compared response to neutral pictures but not during the early stage of perceptual processing. We also hypothesized that chanting may affect concurrent physiological changes given the apparent interaction between emotional stimulus processing and ANS functioning (Critchley and Garfinkel, 2015).

## Materials and Methods

### Participants

Twenty-one participants (10 females) with at least 1 year (approximately 200–3000 h) of experience in chanting the name of Amitābha were recruited for this study. They were all Buddhist, but from different Buddhist sects. The average chanting experience of the participants was 2.1 years and the age of the participants ranged from 40 to 52 years old. Participants with neurological, psychiatric, or other mental disorders were excluded from the study. The consent form approved by (The University of Hong Kong) Human Research Ethics Committee was signed by each participant prior to the experiment. Participants were given $200 HKD for participating in this ERP study.

### Experimental Design

Participants were asked to view pictures depicting negative emotional or neutral content selected from the International Affective Picture System (IAPS) (Bradley and Lang, 2007). The experiment had two factors with a 2 × 3 design. The first factor was the type of picture (neutral or negative) and the second factor was the condition of chanting (chanting the names of either Amitābha or Santa Claus, or no-chanting). This contributed to a total of six combinations of pictures (two types: negative and neutral) and chanting (three conditions: Amitābha, Santa Claus and no-chanting). Based on our pilot data, a block design was chosen since emotional response could be resilient to time; thus, a previous image-induced emotion would inevitably affect the subsequent image-induced emotion, unless the interstimulus interval was set long enough, e.g., about 10 s or more. Thus, we used a block design which could induce a specific emotion in a relatively short time (Stewart et al., 2010).

### Three Chanting Conditions

**FIGURE 1:**
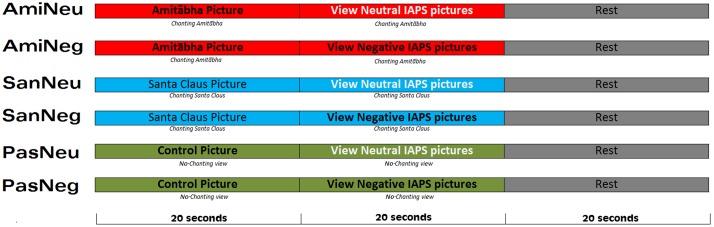
**Experimental procedure follows a 2 × 3 design which includes six conditions.** Each condition was repeated six times and the sequence was pseudorandomized across participants.

The sequence of the six conditions was randomized for each participant. In the chanting Amitābha condition (AMI), participants were asked to chant Amitābha silently while viewing the pictures for 20 s. This procedure was to mimic the real-life practice of chanting Amitābha silently all day long (whenever possible) to train the mind to be focused and equanimous. To help the participants “enter” into a state of chanting during the experiment, they were asked to chant Amitābha silently for 20 s before viewing the pictures. That is, the participants kept chanting Amitābha silently for 40 s (20 s chanting then 20 s chanting plus watching pictures) before taking a rest for 20 s. Thus, a single session lasted for a duration of 60 s. This pattern was the same for all three chanting conditions. See **Figure 1**.

In another condition, participants were asked to chant the name of Santa Claus instead (SAN condition). Santa Claus is a locally well-known figure with whom all participants have been familiar since childhood. We purposely chose such a name because chanting Santa Claus would not elicit negative emotions; rather, we anticipated that participants would either have neutral or slightly positive emotions though adults do not believe Santa Claus to be a real person or a deity. This could somehow control the positive emotion elicited by chanting Amitābha. Moreover, Santa Claus (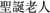) has four Chinese characters, which is the same number of characters as Amitābha (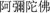). This was designed in an effort to monitor the consistency of pronunciation between conditions. It is to be noted that the participants chanted silently in their minds to avoid muscle movements, which would increase the ERP noise. We also had a no-chanting but passively view condition (PAS), where the participants remained silent without any form of chanting while they passively viewed the pictures. This was set as another control condition, simply differentiating neutral vs. negative pictures.

There were six sessions combined with different picture types/chanting conditions (2 × 3), which were pseudorandomized; i.e., the session sequence and the IAPS pictures were randomized prior to presentation. Different participants had different combinations of picture conditions and chanting conditions to counterbalance the sequence effect. The presentation sequence of the six picture-condition/chanting-condition combinations was counter-balanced across the participants.

### Two Types of Pictures for Viewing

Pictures were all selected from the IAPS. Neutral pictures were those images with neutral facial expressions or everyday objects that would not induce any specific emotion; while negative pictures were gun-aiming and mutilation images^2^ that would provoke fear and stress. The average valence (1 = very unpleasant, 5 = neutral, 9 = very pleasant) for neutral and negative pictures were 5.13 and 2.29; and that the average arousal levels (1 = calm, 9 = excited) for neutral and negative pictures were 3.45 and 6.44. The participants did not rate separately for these images in each of the three chanting conditions, although they did mention slightly feeling easier to confront the negative pictures in the AMI condition. During the experiment, the participants did not make any overt behavioral responses to the pictures, as it may have overlapped with the emotion-related ERP component. Simple behavioral response time usually falls into a similar time course as emotional processing. A post-test recognition task was used to ensure that participants had been attending to the images.

During the picture viewing session, each picture was shown for 1.8–2.2 s, with an inter-stimulus interval of 0.4–0.6 s. Ten pictures of the same condition (neutral or negative) were presented in each block (20 s per block). After the first two blocks (40 s), the third block in that session was a 20-s rest period to counter any potential residual effects of chanting or picture viewing (see **Figure 1** for details).

### Other Experiment Settings

Pictures were presented on a cathode ray tube monitor at a distance of 75 cm from the participant, with a visual angle of 15° (vertical) and 21° (horizontal). Participants were instructed to look at the pictures carefully and a memory test was administered after the experiment. Before the task, each participant performed a brief practice session in order to familiarize them with each condition. There was a video monitor to watch the participants’ performance and confirm that the participants did not fall asleep during the session. None of the participants misunderstood the experiment nor fell asleep during the experiment. After the experiment, each participant was asked to rate their belief in the efficacy of the chanting subject (Amitābha, Santa Claus) using a 1–9 scale, where 1 indicating the least belief and 9 the strongest belief. It showed an average score of 8.16 ± 0.96 for AMI condition, 3.26 ± 2.56 for chanting Santa Claus condition (SAN), and 1.95 ± 2.09 for the passive viewing (PAS) no-chanting condition.

As each of the six picture-condition/chanting condition combinations was repeated six times (about 40 min), with a 20-min rest in the middle, the experiment lasted about 60 min for each participant.

### Data Collection and Analysis

The participants were seated in a dim and quiet room and the experiment was presented using E-prime software (Psychology Software Tools, Inc. USA), which was connected to a 128-channel EGI^TM^ system (Electrical Geodesics, Inc. USA). The impedances of all electrodes remained under 30 KΩ, consistent with the requirements of the EGI system. The sampling rate of the electroencephalography data (EEG) was 1000 Hz. Other physiological data including electrocardiogram (ECG) and GSR were collected by a LabChart^TM^ system (PowerLab, ADInstrument Inc, Australia), following its standard procedures^3^. Three ECG electrodes were placed on the left foot, and on the left and right sides of the waist. Two GSR electrodes were placed on the palm side of the left forefinger and ring finger. The skin was cleaned by alcohol before placement of the electrodes. The GSR value was based on subjective zero, i.e., its value was set as zero at the beginning of the experiment. The physiological data including GSR and ECG were recorded in parallel with E-prime and EEG data.

The EEG data were processed and analyzed with the EEGLab (Delorme and Makeig, 2004) toolbox on the MATLAB^TM^ 11.0 platform (MathWorks Inc. USA). In the preprocessing stage, the data were resampled at 250 Hz, filtered by a finite impulse response filter with a passband of 0.1–100 Hz and notch filtered by a short non-linear infinite impulse response filter with a stopband of 47–53 Hz to reduce any noise caused by 50 Hz mains alternating current. Then, the noisy segments (e.g., body/head movement, obvious muscle artifacts) of the recorded data were deleted manually. Bad channels were reconstructed with spherical interpolation (Carfora, 2007). Consequently, independent component analysis was used to remove the components of eye movement, blinking, and other possible noises. The data were then reconstructed with the retained components.

To obtain the ERP data, the preprocessed EEG data were first filtered by a 30-Hz low-pass filter and then segmented into epochs according to the events and conditions defined by the experiment. EEG epochs with too many bad channels were discarded directly; and epochs with few bad channels would have those channels replaced by spherical interpolation. The data were re-referenced with the average signal of the left and right mastoids. The EEG epochs of each combination of picture type/chanting condition were then averaged as the ERP data. The amplitudes of N1 and LPP were obtained from the second 20 s of a trial. Repeated-measures within-subject ANOVA (Analysis of variance) was used to compare these amplitudes for the N1 and LPP components separately. If the model was significant, *post hoc* analysis (Bonferroni correction) was used to determine the significant differences between two conditions separately. Significance level was set at *p* < 0.05.

Event-related potentials source analysis (Grech et al., 2008) was implemented with the statistical parametric mapping (SPM^4^) the toolbox based on MATLAB (Penny et al., 2011). The coordinate system of the EGI sensor positions was linked to the coordinate system of a standard structural MRI image (MNI coordinates) by landmark-based co-registration. Then, forward computation was performed to calculate the effect of each of the dipoles on the cortical mesh imposed on the EEG sensors. This could result in a matrix G (n × m) where *n* is the number of sensors (EEG space dimension) and *m* is the number of mesh vertices (source space dimension). The source model was *X* = *GS*, where *X* was an n × k matrix denoting the ERP data of each condition, *k* was the number of time points, and *S* was an m × k matrix with elements denoting the signal values of the ERP source. Since the matrix *S* was unknown, the third step was the inverse reconstruction. Among the many different algorithms for inverse reconstruction, we used the Greedy Search-based multiple sparse priors algorithm because it was more reliable than other methods (Friston et al., 2008). The difference between conditions was determined using general linear modeling by SPM. The significance level was set at *p* < 0.05.

The ECG and GSR data were processed and analyzed by powerLab^TM^ and Matlab^TM^. The average scores were obtained for each condition. Similar to the ERP amplitude analysis, the data were further analyzed by repeated-measures ANOVA using SPSS (Statistical Package for the Social Sciences). If the model was significant, *post hoc* analysis (Bonferroni correction) was used to determine the significant differences between the two conditions separately. The significance level was set at *p* < 0.05.

## Results

**FIGURE 2:**
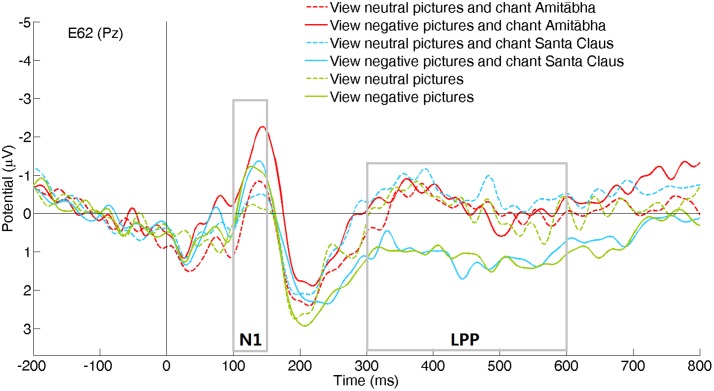
**Event-related potentials (ERPs) recorded at channel Pz for the six picture type/chanting conditions**.

**FIGURE 3:**
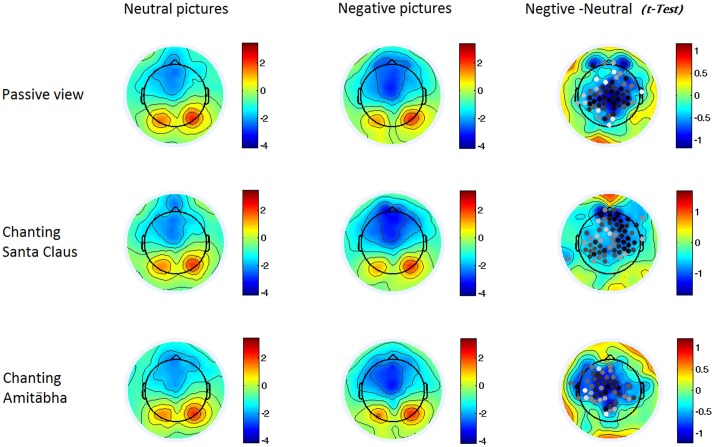
**Two-dimensional maps of the N1 component in the six conditions**. In the last column, the dots indicate channels with significant differences (*p* < 0.05), with darker dots demonstrating smaller *p*-values (i.e., more significance). It indicates that the negative pictures elicited higher negativity around the central regions in all three conditions.

**FIGURE 4:**
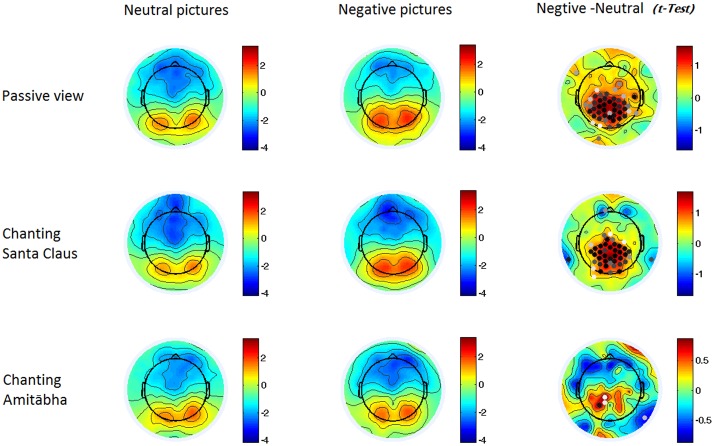
**Two-dimensional maps of the late positive potential component (LPP) in the six conditions for each picture type.** In the last column, the dots indicate channels with significant differences (*p* < 0.05), with darker dots showing smaller *p*-values (i.e., more significance).

The experimental results showed that the chanting conditions had different effects on the early (N1) and late (LPP) processing of negative pictures. The N1 component was not significantly affected by the chanting conditions, while the LPP component was affected by the AMI condition (see **Figures 2–4**). It showed that negative pictures (vs. neutral pictures) elicited higher N1 amplitudes, largely in the central regions by all the three conditions. See **Figures 2** and **3**.

Compared to viewing neutral pictures, negative pictures elicited a higher positive amplitude of LPP in the PAS and SAN conditions, mainly in the centroparietal regions. However, the LPP to negative pictures in the AMI condition had significantly lower amplitude (similar to the LPP to neutral pictures) compared to those in the other two conditions. See **Figures 2** and **4**.

**FIGURE 5:**
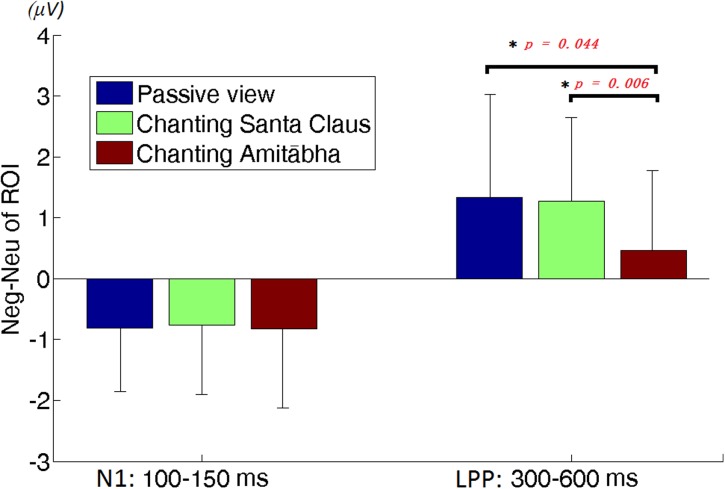
**Region of interest (ROI) analysis on difference of neutral vs. negative pictures elicited amplitudes.** The left three columns are for N1 and the right three columns are for LPP. The error bar is standard deviation. The difference of N1 amplitudes between negative picture and neutral picture (Neg-Neu) in the three conditions is similar. However, the difference of LPP amplitude (Neg-Neu) is significantly lower in the AMI condition than the other two conditions, ^∗^*p* < 0.05.

To better demonstrate the different effect of chanting conditions, two regions of interest (ROI) were selected in the central regions for the N1 and LPP components. To select ROI, the epochs of all three conditions was averaged to calculate those channels where the neutral and negative pictures overall had a significant difference in the specific time window (e.g., for N1 or LPP). **Figure 5** shows the differences between the ERPs for negative pictures and neutral pictures (Neg-Neu, i.e., negative images minus neutral images) in the three conditions. For the N1 component (100–150 ms), repeated-measures ANOVA showed that the effect of chanting (i.e., chanting Amitābha, chanting Santa Claus, passive viewing) was insignificant, *F*(2,40) = 0.19, *p* = 0.981. It indicates that negative pictures similarly elicited larger N1 components than neutral pictures did in all three conditions.

For the LPP component (300–600 ms) ROI analysis, two participants’ data were labeled by boxplot of SPSS^TM^ as outliers and excluded in further data analysis. Repeated-measures ANOVA showed that the main effect of the chanting conditions was significant, *F*(1.46,36) = 4.747, *p* = 0.026 (as sphericity was not assumed, this *p*-value was adjusted by Greenhouse-Geisser for the significance test). The effect size was medium, *d* = 0.514. Further *post hoc* pairwise comparisons (Bonferroni correction) were made between conditions. It demonstrated that the differences between AMI and SAN conditions was highly significant (*p* = 0.006), and the differences between AMI and PAS conditions was significant (*p* = 0.044). However, the difference between SAN and PAS conditions was not significant (*p* = 1.000).

**FIGURE 6:**
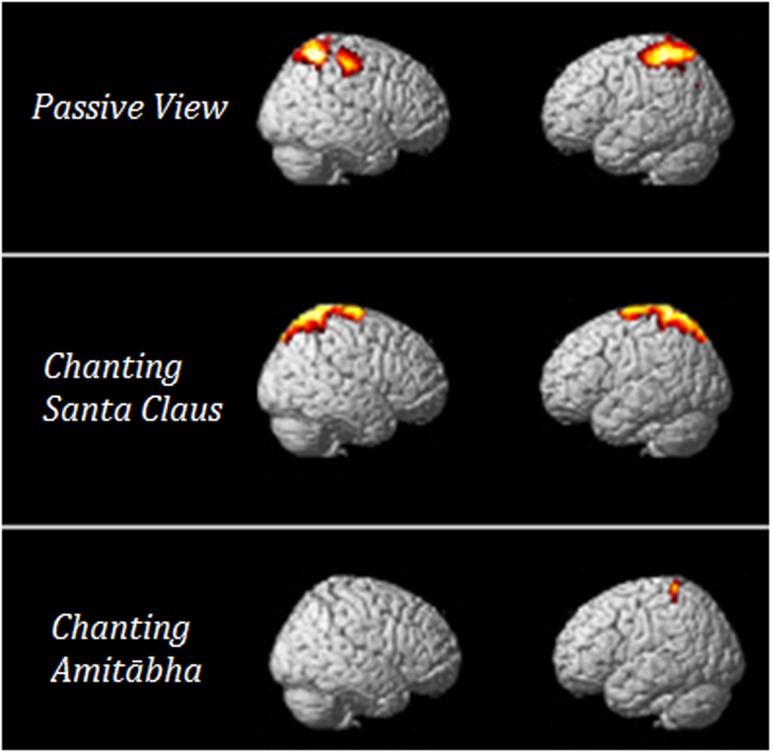
***T*-value of the *t*-test for the source of the LPP under the three conditions.** The results showed that when compared to neutral pictures, negative pictures elicited more parietal activities in the PAS and SAN conditions. These activities could hardly be seen in the AMI condition.

In order to understand which brain regions were most affected by chanting Amitābha, source analysis on the LPP component (300–600 ms) was performed using SPM12. Please also refer to our previous method (Gao et al., 2016). The comparison results are shown in **Figure 6**. It demonstrates that AMI might reduce related brain activity in the parietal lobe, which is involved in late-stage processing on negative pictures.

**FIGURE 7:**
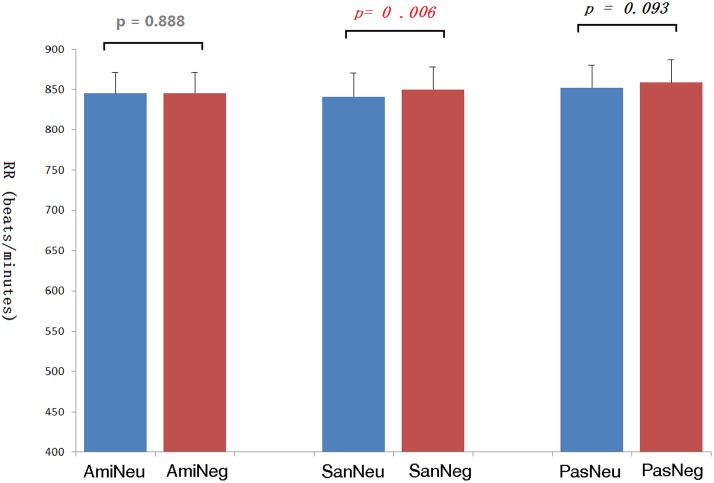
**The inter-beat intervals (RR) of the electrocardiogram under each picture type/chanting combination and the corresponding *p*-values.** Ami, Amifoto chanting condition; San, Santa Claus chanting condition; Pas, passive viewing condition; Neu, neutral picture; Neg, negative picture.

The average inter-beat interval using QRS-QRS (RR) interval of each participant’s ECG was calculated. See **Figure 7**. The repeated-measures ANOVA showed significant differences in the RR due to the effect of picture type, *F*(1,20) = 12.95, *p* = 0.002; and due to chanting conditions, *F*(2,40) = 5.81, *p* = 0.014. Pairwise comparisons with Bonferroni correction showed that compared with no-chanting condition, AMI condition had a significant difference (*p* = 0.038), while SAN condition only had such a trend of difference but not significant (*p* = 0.073). There was no significant difference between AMI and SAN condition (*p* = 1.000). As the effect of picture type on RR was quite significant, further pairwise *t*-tests for RR (**Figure 7**) showed that the heart rate was slower when viewing negative pictures in the PAS and SAN conditions (*p* = 0.093 and *p* = 0.001, respectively, Bonferroni corrected). However, during the AMI condition, the heart rate intervals when viewing neutral and negative conditions were similar (*p* = 1.000). We further calculated the heart rate variability, i.e., standard deviation of normal to normal (SDNN). There was no difference between the three chanting conditions, *F*(2,40) = 0.630, *p* = 0.680.

The mean GSR of each picture type/chanting combination^5^ was as follows: AmiNeu (1.339 ± 1.785 μS), AmiNeg (1.348 ± 1.789 μS), SanNeu (1.321 ± 1.772 μS), SanNeg (1.376 ± 1.821 μS), PasNeu (1.290 ± 1.751 μS), and PasNeg (1.303 ± 1.776 μS), respectively. The repeated-measures ANOVA indicated that the GSR was not significantly different among the conditions; *F*(5,20) = 1.37, *p* = 0.242. The mean of respiratory rate interval was: AmiNeu (3.282 ± 0.351 s), AmiNeg (3.318 ± 0.293 s), SanNeu (3.317 ± 0.249 s), SanNeg (3.311 ± 0.354 s), PasNeu (3.430 ± 0.318 s), and PasNeg (3.354 ± 0.336 s). There was no any difference among the conditions; *F*(5,20) = 1.37, *p* = 0.242.

## Discussion

Chanting and prayer are the most popular religious practices and many religious devotees believe that these practices would help them to live through hardships. To delineate its neural mechanism, we used ERP to precisely investigate the influence of religious chanting on the brain’s response to negative events. It turned out that chanting Amitābha could reduce the individual’s emotional response at the late-stage, but not at the early-stage processing of negative events. This finding confirms our hypothesis that the processing of negative information, religious practice such as chanting would not affect the early neural processing of N1 but only affect the late-stage processing of LPP.

To better understanding these findings, we want to know if the participants had employed the distraction strategy (Gross, 1998; Thiruchselvam et al., 2011) by shifting their attention away from the negative pictures as some of these pictures were quite fear-provoking and stress-provoking, and even aversive. Furthermore, the participants might have also become more involved in chanting Amitābha and were distracted from watching the negative images even at an early perceptual stage. The early component of ERP showed that this was not the case. The initial perceptual response (N1 component) to negative pictures was higher than the response to neutral pictures in all three chanting conditions. This demonstrates that the participants were attending to the negative pictures, but not avoiding them in the AMI condition. Actually, the N1 amplitude to negative pictures was slightly higher in the AMI condition than in the other two conditions, although the difference was not significant.

The visual N1 component is a large, central negative deflection that peaks at about 130 ms after picture onset (Foti et al., 2009). It mainly represents sensory information processing in the early stage and its amplitude varies with attention selectively paid to the stimuli. The N1 relies more on the stimuli’s physical features and thus is more resistant to habituation, especially for unpleasant pictures (Carretie et al., 2003). The negative pictures elicited a higher N1 component, and it was comparable in all the three conditions. A higher N1 amplitude reflects more intensive visual processing of the emotional content of negative pictures even at a very early stage (Coull, 1998). Our finding of a higher N1 response to emotional content is consistent with previous studies demonstrating that negatively valenced pictures can raise arousal levels and deplete more attentional resources than neutral pictures (Foti et al., 2009). N1 is also attributed to the processing communication between the visual cortex and the dorsolateral frontal cortex (Foxe and Simpson, 2002). Our results indicate that N1 was quite robust and was not influenced by any of the chanting conditions. Chanting the names of different figures with quite different beliefs could not affect the N1 component.

In the later stages of information processing, negative pictures elicited a significantly larger LPP in the PAS and SAN conditions. The higher LPP component is typically found in response to negative pictures in previous ERP studies. For example, De Cesarei et al. found that highly arousing negative pictures evoked a greater LPP than highly arousing positive pictures did (De Cesarei and Codispoti, 2006). The LPP is responsible for recognizing and categorizing the stimuli (Codispoti et al., 2006). Negative, especially fear-provoking and stress-provoking events/pictures, may activate the relevant neural circuits such as hypothalamic autonomic nuclei responsive for orienting and action. This processing is mandatory due to its evolutionary significance, and it is associated with the motivational systems in the brain for defensive or appetitive responses (Lang et al., 1997).

Interestingly, when participants were chanting Amitābha, the effect of the negative pictures on LPP has largely disappeared and became similar to those responses to neutral pictures. Given that LPP would remain robust even during tedious repetition of stimuli (De Cesarei and Codispoti, 2006), this counterbalancing effect on the response to negative pictures when chanting Amitābha was quite prominent, when compared to the other two conditions of no-chanting and chanting of Santa Claus.

Source analysis showed that negative images during the period of LPP elicited greater neural activity in the central-parietal regions of the brain in the PAS and SAN conditions, but only minimal neural activities were observed in the AMI condition. The LPP is associated with the motivational systems in the brain (i.e., the defensive/appetitive systems), which in turn activate the relevant neural circuits that are responsive to orientation and action (Lang et al., 1997). The parietal lobe is involved in emotion regulation (Etkin et al., 2015; Rice, 2016). Previous studies have observed that the parietal lobe is responsive to orientation and visuomotor transformation for actions (Murata et al., 2000; Fogassi and Luppino, 2005). Less activation in the relevant brain areas may imply that when chanting Amitābha, the participants were less reactive.

One preliminary single-photon emission computed tomography study on meditative prayer found that repeating a particular phrase from the Bible can change the blood flow in the frontal and parietal regions (Newberg et al., 2003). By the same token, it is plausible that chanting Amitābha may cause the individual’s brain to be less motivated to act or react to negative events. Similarly, Koole et al. (2010) found that prayer could consistently ameliorate negative moods. It is argued that religiosity can lift the emotional burden by implicit self-regulation.

The tendency to focus heavily on negative information can be reduced by mindful breathing (Kiken and Shook, 2011); chanting Amitābha may also help the participants to be more mindful of their surroundings and to have some control over their physiological responses to external stimuli. Our study is in line with previous research that mindfulness can reduce the automaticity of response biased to age and race (Lueke and Gibson, 2014). According to Dharma Therapy, mindfulness may be more powerful if integrated with other components of Buddhist teachings and practices (Sik, 2011). Verbal repetitions of a sequence of a particular tune or the vibration of sound may be utilized as contemplative aids for acquiring attentiveness, presence of mind, and for triggering a series of positive associations through correlative thinking that links the name of Amitābha Buddha with symbolic and literary narratives of his Pure Land Sukhâvatî, literally the “land of bliss” (Halkias, 2013a). Similarly, the broaden and build theory of positive emotion, positive emotion can help to broaden and build a momentary thought-action repertoire (Fredrickson, 2004).

According to previous research, practicing Catholics had lower pain ratings when watching the image of the Virgin Mary. It is suggested that detachment strategy enhanced by the religiously positive image could help the participants detach from an immediate negative experience (Wiech et al., 2008). Furthermore, belief in an omnibenevolent or omnipotent divine agent fosters appraisals and helps individuals to build up resilience against stressful experiences (Vishkin et al., 2016). The habit of chanting Amitābha might help to a form a religious schema that has similar effect. From the above discussion on Christian-faith based researches and our own findings on the effect of Buddhist chanting, prayer/religious practices may have cross-cultural universality in emotion-regulatory significance.

As to the physiological measurements, the ECG data showed that the participants’ heart rates were lower when viewing negative pictures as compared to that when viewing neutral pictures in the SAN condition; there was a similar trend in the PAS condition. A slower heart rate indicates a more parasympathetic nervous system activity (Taylor, 1991). As our negative pictures from IAPS included some mutilation scenes, it might induce defensive bradycardia (Mocaiber et al., 2011). It has also been shown that an automatically offsetting physiological response (e.g., lower heart rate) to arousal will occur as a compensatory process to negative stimuli, especially fear-provoking pictures (Bornas et al., 2006). However, this phenomenon disappeared in the AMI condition. Our results imply that chanting Amitābha could offset this effect to some extent.

The GSR measurement did not show significant difference between conditions; this may be partly due to that the GSR could rapidly decline after the repetition of IAPS negative pictures (Codispoti et al., 2006). We further analyzed the first part of the GSR of all sessions; yet still did not find any difference. Using emotional visual/auditory stimuli, Brouwer et al. (2013) found a decelerated heart rate to high-arousal negative stimuli, which is consistent with current findings. However, they also found increased heart rate variability (HRV) and GSR. One possible reason could be that they used a much long blocks, about 2 min, while we used 20-s blocks. A longer duration might enable a more reliable measurement on physiological change with emotion. This should be considered in future experimental designs. However, longer duration may lead to participant fatigue.

Our EEG and part of the physiological data imply that chanting or other popular religious practices, including mindfulness, may indeed help individuals reduce distress and/or prevent emotional reactivity when confronted with negative affect-producing stressors (Kiken and Shook, 2012; Labelle et al., 2015). These findings echo the previous findings of a research on watching the image of the Virgin Mary by providing evidence that Buddhist chanting, or in a broader sense, repetition of religious practice, can also modulate brain responses to negative stimuli (Wiech et al., 2008). As have already stated above, prayer/religious practices may have cross-cultural universality in emotion-regulatory significance. In addition, our findings show for the first time that Buddhist chanting, or in a broader sense, repetition of religious practice, can modulate brain responses to negative stimuli not during the early perceptual stage but during late-stage emotional/cognitive processing.

These findings from both brain and physiological response partially confirm with our hypothesis based on the *Sallatha Sutta* (*The Arrow Sutra*, Bhikkhu, 2013) that a well-trained practitioner would experience the suffering of the first arrow only but not the second one. The current study gives objective evidence to the widely adapted position that key components of religious life such as prayers, rituals, music, etc., which may invoke some kind of unique emotion of the believers, can partly modulate the tendency of a negative response to difficulties in real life (Corrigan, 2008).

There are several limitations in this research. First of all, the familiarity of chanting the name of Amitābha vs. the unfamiliarity of chanting Santa Claus could not be controlled. It is difficult to conclude at this stage whether chanting any other name with greater familiarity would have had a similar effect. Furthermore, the heart rate usually increases when confronting with fear-provoking pictures, while our result showed a decrease in heart rate. It could be our negative pictures included mutilation images from IAPS, which could induce defensive bradycardia (Mocaiber et al., 2011). We need a more refined experimental design and probably other functional neuroimaging to differentiate this important issue. Clarification of these neural mechanisms may be the basis of alternative methods for improving psychological resilience.

## Ethics Statement

Human Research Ethics Committee (HREC), The University of Hong Kong approved the ethical application of this study. After the approval, we began to recruit participants for EEG experiment. Before the experiment, we explained the procedure to them, and they were told about the experiment and they could withdraw from the experiment at any time. Written consent from was signed by each participant before she/he did the experiment. The participant was thanked for their effort in the experiment and was given transportation in review fee.

## Author Contributions

JG helps to design the experiment, write up the initial manuscript. JF helps to do the experiment, BW, GH, and MC help to make revisions on the manuscript. PF helped to design the experiment and revision. CC, ZZ, and Y-SH help to design the experiment and give suggestion on data analysis. HS helps to design the experiment, make revisions on manuscript.

## Conflict of Interest Statement

The authors declare that the research was conducted in the absence of any commercial or financial relationships that could be construed as a potential conflict of interest.

## Footnotes

1 This Buddhist employment of “vocal formulas and sacred names” resembles the widespread use of dharaṇī in Mahayana Buddhism and bears resemblance to “talismanic invocations,” “magic scripts and formulas,” and the “pirit ceremony” in Śrāvakayāna traditions of Buddhism.

2 Neutral pictures included IAPS 2221, 2230, 2372, 2381, 2383, 2410, 2440, 2514, 2516, 2518, 2520, 2590, 2600, 2880, 2890, 5460, 5740, 5875, 7002, 7170, 7184, 7217, 7495, 7496, 7500, 7550, 7700, 7705, 7900, 7950. Negative pictures included IAPS 3030, 3168, 3170, 3266, 3301, 3500, 3530, 6190, 6211, 6212, 6230, 6243, 6244, 6313, 6350, 6370, 6510, 6540, 6550, 6560, 6570, 6571, 6821, 6834, 9050, 9405, 9410, 9570, 9600, 9620.

3 https://www.adinstruments.com/support/manuals

4 http://www.fil.ion.ucl.ac.uk/spm

5 Chanting conditions: Ami, Amitābha; San, Santa Claus; Pas, passive view. Picture types: Neu, neutral picture; Neg, negative picture.
